# Efficacy and safety of B7-H3-targeted antibody-drug conjugates in previously treated small cell lung cancer: a systematic review and meta-analysis with a contextual comparison to topotecan

**DOI:** 10.3389/fimmu.2026.1907324

**Published:** 2026-08-13

**Authors:** Jiahui Wang, Hongqin Jiang, Junzi Niu, Lixia Liu, Yijun Wu, Jianlin Shi, Liya Liu, Shouyan Yin, Chun Feng, Lei Zhang, Mei Yang, Zihan Yi

**Affiliations:** 1 Phase I Clinical Trial Ward, The Third Affiliated Hospital of Kunming Medical University, Yunnan Cancer Hospital, Peking University Cancer Hospital Yunnan, Kunming, China; 2 Department of Biochemistry and Molecular Biology, School of Basic Medicine, Kunming Medical University, Kunming, China; 3 Faculty of Pharmacy, Kunming Medical University, Kunming, China; 4 Department of Chest Surgery, The Third Affiliated Hospital of Kunming Medical University, Yunnan Cancer Hospital, Peking University Cancer Hospital Yunnan, Kunming, China; 5 Department of Otolaryngology, The First People’s Hospital of Yunnan Province, The Affiliated Hospital of Kunming University of Science and Technology, Kunming, China; 6 Department of Gynecology, The Third Affiliated Hospital of Kunming Medical University, Yunnan Cancer Hospital, Peking University Cancer Hospital Yunnan, Kunming, China

**Keywords:** antibody-drug conjugates, B7-H3, efficacy, safety, small cell lung cancer, topotecan

## Abstract

**Objective:**

B7-H3-targeted antibody-drug conjugates (B7-H3-targeted ADCs) have shown promising antitumor effects in patients with previously treated small cell lung cancer (SCLC), yet the available evidence on their efficacy and safety has not been systematically synthesized. This study aimed to systematically evaluate the efficacy and safety of B7-H3-targeted ADCs and contextualize their clinical outcomes against standard-dose intravenous topotecan.

**Methods:**

This systematic review and meta-analysis was conducted in accordance with PRISMA guidelines. PubMed, Embase, and the Cochrane Library were searched from inception to July 7, 2026. Prospective single-arm studies of B7-H3-targeted ADCs and randomized controlled trials (RCTs) with separately extractable topotecan arms were included. Study quality was assessed using the Newcastle–Ottawa Scale and Cochrane RoB 2 tool. Random-effects models were used to pool efficacy and safety outcomes. Evidence from the two treatment groups was synthesized separately. Results for B7-H3-targeted ADCs were descriptive, whereas topotecan was used solely as an external clinical benchmark; therefore, the contextual comparison should not be interpreted as a direct comparative estimate.

**Results:**

Ten studies were included, comprising four prospective single-arm studies of B7-H3-targeted ADCs and six RCTs with eligible topotecan arms. For B7-H3-targeted ADCs, the pooled objective response rate (ORR) and disease control rate (DCR) were 54% (95% CI: 46%–62%) and 89% (95% CI: 85%–92%), respectively. The pooled median duration of response and median progression-free survival were both 5.6 months (95% CI: 4.8–6.4 and 4.7–6.5 months, respectively). The pooled incidences of any-grade and grade ≥3 treatment-related adverse events were 99% (95% CI: 98%–100%) and 61% (95% CI: 55%–67%), respectively. In the exploratory contextual analysis, ifinatamab deruxtecan achieved an ORR of 49% (95% CI: 41%–57%) and a DCR of 87% (95% CI: 81%–92%), whereas the corresponding estimates for topotecan were 18% (95% CI: 15%–22%) and 65% (95% CI: 61%–69%). Overall, the exploratory comparison showed numerically higher efficacy estimates and selected safety differences favoring ifinatamab deruxtecan compared with topotecan.

**Conclusions:**

B7-H3-targeted ADCs show promising clinical activity in patients with previously treated SCLC. In the exploratory contextual comparison, ifinatamab deruxtecan showed numerically favorable efficacy estimates and selected safety outcomes compared with topotecan.

**Systematic review registration:**

https://www.crd.york.ac.uk/PROSPERO, identifier CRD420251237597, version 3.0.

## 1 Introduction

Cancer remains a major cause of mortality worldwide, and its incidence continues to rise annually (1). In China, lung cancer is one of the most common forms of cancer and a leading cause of cancer-related mortality (2). Small cell lung cancer (SCLC) represents a particularly aggressive form of lung cancer that is often diagnosed at an advanced stage (3). In recent years, the introduction of immune checkpoint inhibitors has substantially reshaped the treatment landscape of extensive-stage SCLC (ES-SCLC), establishing platinum-etoposide plus an inhibitor of programmed cell death protein 1 or programmed death-ligand 1, followed by maintenance immunotherapy, as a standard first-line treatment (4–6). However, most patients eventually experience disease progression. Historically, subsequent-line treatment has relied largely on agents such as topotecan, lurbinectedin and amrubicin, with limited durable clinical benefit (7). More recently, tarlatamab, a bispecific T-cell engager directed against delta-like ligand 3 (DLL3), demonstrated a significant overall survival benefit over investigator’s-choice chemotherapy in the phase III DeLLphi-304 trial and has emerged as a new second-line standard of care (8). Despite this important advance, substantial unmet clinical needs remain in relapsed SCLC. The development of therapeutic strategies with distinct targets and mechanisms of action therefore remains a priority, with antibody-drug conjugates (ADCs) emerging as an important area of drug development in SCLC.

ADCs are a class of targeted anticancer agents, each comprising a monoclonal antibody conjugated to a potent cytotoxic payload via a linker (9). The antibody component recognizes tumor-associated antigens and facilitates preferential delivery of the payload to malignant cells (10). However, despite this tumor-directed design, ADCs remain systemically administered agents, and normal-tissue exposure to the ADC and systemic exposure to the released payload may contribute to toxicity (11). The linker is essential for preserving molecular stability in the circulation and enabling efficient payload release after internalization into tumor cells (12). The payload serves as the principal effector, mediating direct tumor cell killing (13). After antigen recognition, ADCs are internalized and processed intracellularly, leading to payload release, interference with key cellular pathways and, in some settings, modulation of the tumor immune microenvironment (14, 15). In SCLC, several tumor-associated antigens have been investigated as ADC targets, including B7 homolog 3 (B7-H3, also known as CD276), DLL3, trophoblast cell surface antigen 2 (TROP-2), seizure-related homolog 6 (SEZ6), and neural cell adhesion molecule 1 (also known as CD56) (16, 17).

B7-H3 is a member of the B7 family of molecules that regulate the immune system. The components of the B7/CD28 pathway play a crucial role in maintaining immune balance, as they provide both activating and suppressive signals that influence immune responses against tumors (18). Unlike some other B7 family members, B7-H3 appears to exert context-dependent effects, with its biologic role varying according to the immune cell population involved, features of the tumor microenvironment, and the influence of nonimmune signaling pathways (19, 20). In SCLC, B7-H3 protein expression is detectable in approximately 65% of cases (21), whereas its expression in normal tissues is relatively limited. A recent analysis showed that B7-H3 RNA expression is high and relatively consistent across the four major molecular subtypes of SCLC—SCLC-A, SCLC-N, SCLC-P and SCLC-I—suggesting that its potential as a therapeutic target may extend beyond any single molecular subtype (22). Preclinical studies across tumor models further suggest that B7-H3 may contribute to immune evasion by modulating T-cell and natural killer cell function and may also promote tumor progression through nonimmune mechanisms (23–25). Together, these features provide a biological rationale for developing B7-H3-targeted therapies in SCLC, particularly B7-H3-targeted ADCs.

Currently, clinical evidence for B7-H3-targeted ADCs in SCLC remains at an early stage and is derived predominantly from phase I/II single-arm trials, with no randomized controlled trials (RCTs) directly comparing these agents with established treatments for previously treated SCLC. Although these studies suggest promising antitumor activity, the clinical benefit and safety profile of B7-H3-targeted ADCs relative to existing later-line treatment options require systematic evaluation. Topotecan is a semisynthetic camptothecin analogue and topoisomerase I inhibitor that disrupts DNA replication, resulting in lethal DNA double-strand breaks and tumor cell death (26, 27). Topotecan has long been a conventional treatment option for patients with relapsed or refractory SCLC and has served as a control regimen in multiple RCTs evaluating emerging therapies in this setting (28, 29). Despite the emergence of new second-line options (30), the extensively documented efficacy and safety of standard-dose intravenous (IV) topotecan make it a well-characterized clinical benchmark. Accordingly, the present systematic review and meta-analysis synthesizes the available efficacy and safety evidence for B7-H3-targeted ADCs in previously treated SCLC and, where sufficient data are available, provides an exploratory contextual comparison of ifinatamab deruxtecan (I-DXd) with standard-dose IV topotecan. This study aims to provide insights into the design of future RCTs of B7-H3-targeted ADCs, including the selection of efficacy endpoints, safety monitoring, and optimization of combination treatment strategies.

## 2 Methods

### 2.1 Search strategy

This systematic review and meta-analysis was reported in accordance with the Preferred Reporting Items for Systematic Reviews and Meta-Analyses 2020 statement (PRISMA 2020) (31, 32). The review protocol was prospectively registered in the International Prospective Register of Systematic Reviews (PROSPERO; CRD420251237597), and subsequent amendments were documented in Version 3.0. PubMed, Embase, and the Cochrane Library were searched from database inception to July 7, 2026. Controlled vocabulary and free-text terms for SCLC were combined with intervention-specific terms for B7-H3/CD276-targeted ADCs or topotecan to maximize retrieval sensitivity. The reference lists of relevant articles and reviews were also screened manually to identify additional eligible studies. The complete search strategy is provided in **Supplementary Table 1**.

### 2.2 Inclusion and exclusion criteria

Studies were eligible if they met all of the following criteria (1): enrolled adults aged ≥18 years with histologically or cytologically confirmed SCLC that had relapsed or progressed after at least one prior platinum-based systemic therapy (2); evaluated B7-H3-targeted ADC monotherapy or standard-dose IV topotecan monotherapy (1.5 mg/m² on days 1–5 every 21 days) (3); used a prospective interventional design, including prospective single-arm studies for B7-H3-targeted ADCs and RCTs with separately extractable data from the eligible topotecan monotherapy arm (4); reported at least one analyzable efficacy or safety outcome, including objective response rate (ORR), disease control rate (DCR), median duration of response (mDOR), median progression-free survival (mPFS), median overall survival (mOS), or adverse events (AEs); and (5) were full-text original articles, with no language restrictions. Studies were excluded if data for the SCLC population or treatment arm of interest could not be separately extracted; if they were retrospective studies, reviews, case reports, conference abstracts, protocols, letters, or other non-original publications; or if they reported duplicate or overlapping populations. The detailed eligibility criteria are presented in **Table 1**.

**Table 1:** List of key eligibility criteria for studies to be included.

Criteria	Included	Excluded
Population	• Adults (≥18 years) with histologically or cytologically confirmed small cell lung cancer. • Patients with relapsed or progressive disease after ≥1 prior line of platinum-based systemic therapy.	• Patients aged <18 years. • Non-small cell lung cancer or other tumor types. • Studies without separately extractable SCLC-specific data.
Intervention	• B7-H3-targeted antibody–drug conjugate monotherapy.	• Combination regimens or studies without separately extractable B7-H3-targeted ADC monotherapy data.
External clinical benchmark	• Standard-dose intravenous topotecan monotherapy (1.5 mg/m² on days 1–5 of each 21-day cycle).	• Oral topotecan, combination regimens, and non-standard doses or administration schedules.
Study design	• Prospective interventional clinical studies, including single-arm studies for B7-H3-targeted ADCs and randomized controlled trials for the topotecan benchmark. • Full-text original articles.	• Retrospective studies. • Reviews, case reports, conference abstracts, comments, and editorials. • Duplicate reports.
Language	• No language restrictions.	• None
Date	• From database inception to July 7, 2026.	• None

### 2.3 Study selection and data extraction

Two reviewers independently conducted study selection in two sequential stages according to the predefined eligibility criteria. Titles and abstracts were screened first, after which the full texts of potentially eligible reports were independently evaluated for final inclusion. The same reviewers independently extracted data from each included study using a standardized and prespecified form. Any disagreement arising during study selection or data extraction was resolved through discussion and, when necessary, adjudication by a third reviewer. Extracted variables included the first author, publication year, country, study design, trial phase, tumor histology, treatment regimen, patient age and sex, and the reported efficacy and safety outcomes.

### 2.4 Risk of bias assessment

The methodological quality of the included studies was independently evaluated by two reviewers. Prospective nonrandomized B7-H3-targeted ADC studies were assessed using the NOS, whereas the included RCTs were evaluated using RoB 2. Any disagreement between the reviewers was resolved through discussion until consensus was reached.

### 2.5 Data synthesis and analysis

In the absence of RCTs or prospective studies directly comparing B7-H3-targeted ADCs with standard-dose IV topotecan, evidence for the two treatment modalities was analyzed separately. Topotecan was treated exclusively as an external clinical benchmark, and the contextual analysis was restricted to I-DXd when sufficient data were available. All analyses were conducted using R version 4.4.1 with the meta package. ORR, DCR, and safety endpoints were summarized as pooled proportions with corresponding 95% CIs using the Freeman–Tukey double-arcsine transformation under a random-effects framework. Between-study variance was estimated using the restricted maximum-likelihood method. Reported mDOR, mPFS, and mOS estimates, together with their corresponding 95% CIs, were synthesized using generic inverse-variance models. Statistical heterogeneity was quantified using I² and τ² (32). Random-effects models were used because heterogeneity was anticipated. When only one study reported an outcome, the study-specific estimate was presented descriptively rather than pooled. No cross-treatment pooled estimate, indirect treatment-effect estimate, or formal between-group hypothesis test was generated.

### 2.6 Deviations from protocol

The original registered protocol specified a broad comparator (“usual care”) and planned to include only nonrandomized studies. Because no direct comparative studies of B7-H3-targeted ADCs versus conventional later-line treatments were identified, standard-dose IV topotecan was selected as an external clinical benchmark. Topotecan is a well-characterized conventional treatment for relapsed SCLC and has been evaluated in multiple prospective RCTs reporting efficacy and safety endpoints analogous to those available for B7-H3-targeted ADCs. Topotecan was used solely for an exploratory contextual comparison rather than as a direct control group. RCTs were therefore eligible only when data from the standard-dose IV topotecan monotherapy arm could be extracted separately; these arm-level data were not used to estimate randomized comparative effects. The original protocol prespecified the Risk Of Bias In Non-randomized Studies of Interventions tool and comparative effect measures, including risk ratios, mean differences, and hazard ratios. Because the B7-H3-targeted ADC evidence comprised prospective single-arm studies without comparator groups, the NOS was used to assess these studies, whereas RoB 2 was applied to the topotecan trials according to their original randomized design. In the absence of direct comparative evidence, outcomes were synthesized separately using arm-based estimates and interpreted descriptively. The prespecified certainty-of-evidence assessment using the Grading of Recommendations Assessment, Development and Evaluation framework was not undertaken because no corresponding direct comparative estimates were generated. Publication bias was not formally assessed because fewer than 10 studies contributed to any pooled estimate, resulting in limited power for funnel-plot asymmetry tests. These amendments are documented in PROSPERO Version 3.0.

## 3 Results

### 3.1 Study selection and characteristics

**Figure 1:**
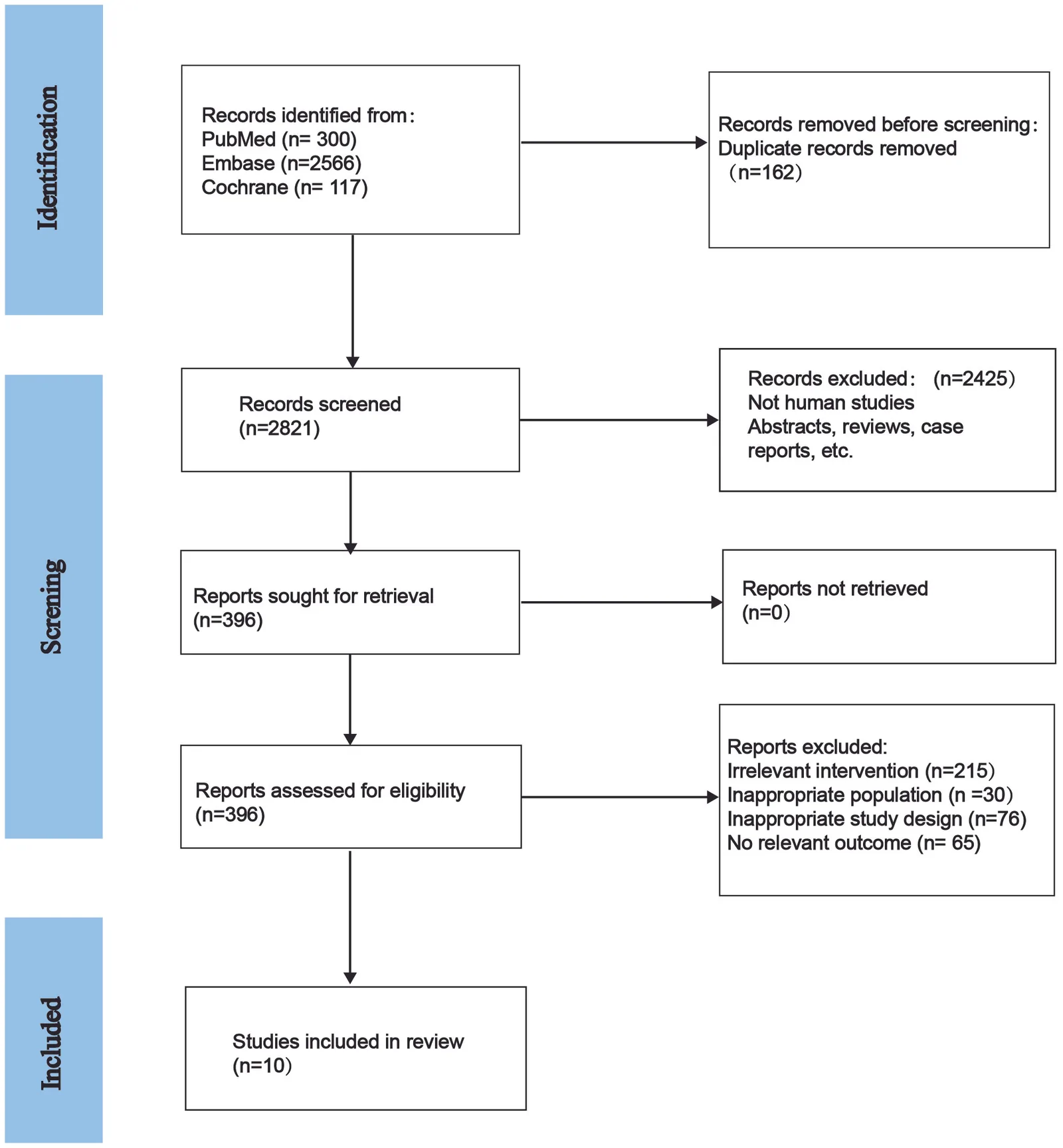
PRISMA flow diagram.

Searches of PubMed, Embase, and the Cochrane Library identified 2,983 records. The study selection process is presented in the PRISMA 2020 flow diagram (**Figure 1**). After removal of 162 duplicate records, 2,821 records underwent title and abstract screening, of which 2,425 were excluded. The full texts of all 396 potentially eligible reports were retrieved; none was unavailable. Following full-text assessment, 386 reports were excluded because of an irrelevant intervention (n = 215), an ineligible population (n = 30), an ineligible study design (n = 76), or no extractable outcome data (n = 65). Ten studies were ultimately included in the systematic review and meta-analysis, comprising four studies of B7-H3-targeted ADCs (33–36) and six studies of standard-dose IV topotecan (28, 37–41). Potentially relevant B7-H3-targeted ADC studies that were not included are detailed, together with the reasons for exclusion, in **Supplementary Table 2**. The characteristics of the included studies are summarized in **Table 2**. All four B7-H3-targeted ADC studies were single-arm trials: one phase I/Ib trial, one phase Ia/Ib trial, one phase I/II trial, and one phase II trial. The topotecan studies comprised two phase II trials, one phase IIb trial, and three phase III trials. Across the included studies, the median age ranged from 57.0 to 68.0 years, and male patients accounted for the majority of participants.

**Table 2:** Characteristics of the included studies.

Study ID	Trial number	Study design	Phase of the study	Intervention	Age(years)	N(M/F)
Rudin-2025	NCT05280470	single-arm	II	Ifinatamab deruxtecan, 12 mg/kg, IV, Q3W	63 (34–79)	90/47
Ma-2025	NCT05434234; NCT06057922	single-arm	I/Ib	YL201, 2.0 mg/kg or 2.4 mg/kg, IV, Q3W	61 (45–76)	68/11
Duan-2026	NCT05276609	single-arm	Ia/b	HS-20093, 8.0 mg/kg or 10.0 mg/kg, IV, Q3W	57 (30–74)	53/16
Johnson-2026	NCT04145622	single-arm	I/II	Ifinatamab deruxtecan, ≥6.4 mg/kg, IV, Q3W	61 (57–69)	14/8
Jotte-2011	NCT00319969	RCT	II	Topotecan, 1.5 mg/m², IV, d1-5, Q3W	68 (46–84)	11/15
Pawel-2014	NCT00547651	RCT	III	Topotecan, 1.5 mg/m², IV, d1-5, Q3W	61 (30–81)	127/86
Evans-2015	NCT01500720	RCT	II	Topotecan, 1.5 mg/m², IV, d1-5, Q3W	62 (27–80)	NR
Kang-2021	NCT01497873	RCT	IIb	Topotecan, 1.5 mg/m², IV, d1-5, Q3W	67 (42–83)	62/14
Edelman-2022	NCT03098030	RCT	III	Topotecan, 1.5 mg/m², IV, d1-5, Q3W	62 (34–84)	68/26
Spigel-2024	NCT03088813	RCT	III	Topotecan, 1.5 mg/m², IV, d1-5, Q3W	62 (28–81)	163/69

### 3.2 Quality assessment

**Figure 2:**
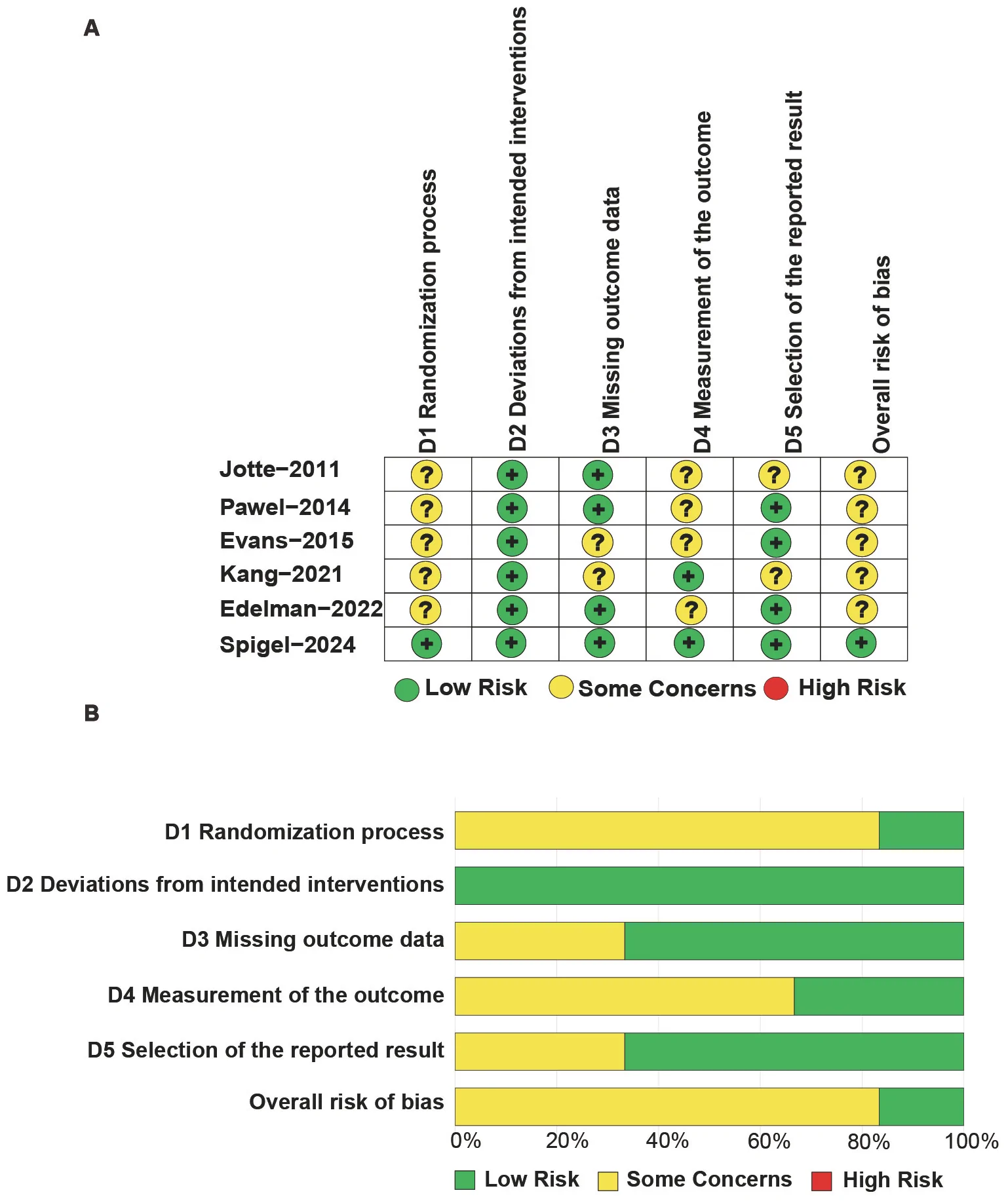
Quality assessment of the randomized controlled trials. **(A)** Details of the bias assessment. **(B)** Summary of the bias assessment.

Risk-of-bias assessments are summarized in **Table 3** and **Figure 2**. All four single-arm studies received favorable ratings across the applicable NOS domains (**Table 3**). According to the RoB 2 assessment, one RCT was judged to have a low overall risk of bias, whereas the other five RCTs raised some concerns; no RCT was judged to have a high risk of bias. The most common concerns related to the randomization process, with additional concerns in selected studies regarding missing outcome data, measurement of the outcome, or selection of the reported result (**Figure 2**).

**Table 3:** Risk of bias assessment table for single-arm studies.

Risk of bias assessment									
Study/year		Selection				Comparability			
Ref ID	Study	(1)Representativeness of the exposed cohort	(2)Selection of the non-exposed cohort	(3)Ascertainment of exposure	(4)Demonstration that the outcome of interest was not present at the start of the study	(1)Comparability of cohorts based on the design or analysis	(2)Assessment of outcome	(3) Was the follow-up long Enough for Outcomes to occur	(4)Adequacy of follow-up of cohorts
1	Rudin-2025	Yes	NA	Yes	Yes	NA	Yes	Yes	Yes
2	Ma-2025	Yes	NA	Yes	Yes	NA	Yes	Yes	Yes
3	Duan-2026	Yes	NA	Yes	Yes	NA	Yes	Yes	Yes
4	Johnson-2026	Yes	NA	Yes	Yes	NA	Yes	Yes	Yes

### 3.3 Efficacy outcomes of B7-H3-targeted ADCs

**Figure 3:**
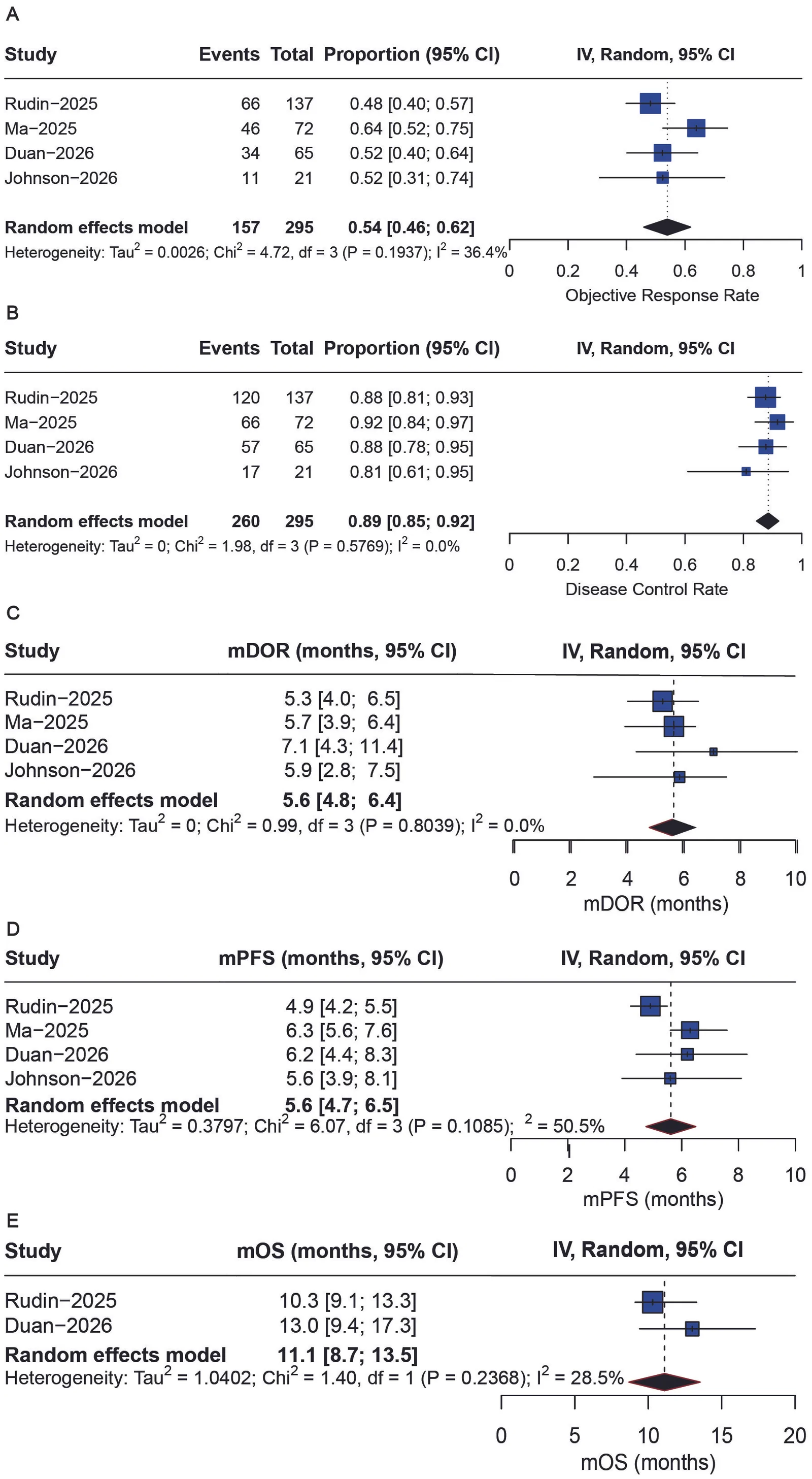
Forest plots of efficacy outcomes of B7-H3-targeted ADCs. **(A)** ORR; **(B)** DCR; **(C)** mDOR; **(D)** mPFS; **(E)** mOS.

Four studies comprising 295 efficacy-evaluable patients contributed to the analyses of ORR, DCR, mDOR, mPFS and mOS. Using a random-effects model, the pooled ORR was 54% (95% CI: 46%–62%, I² = 36.4%, *P*=0.1937) (**Figure 3A**). The pooled DCR was 89% (95% CI: 85%–92%, I² = 0.0%, *P*=0.5769) (**Figure 3B**). The pooled mDOR was 5.6 months (95% CI: 4.8–6.4 months, I² = 0.0%, *P*=0.8039) (**Figure 3C**). The pooled mPFS was 5.6 months (95% CI: 4.7–6.5 months, I² = 50.5%, *P*=0.1085) (**Figure 3D**), with moderate between-study heterogeneity. mOS data were available from two studies (Rudin-2025 and Duan-2026) comprising 202 patients. The pooled mOS was 11.1 months (95% CI: 8.7–13.5 months, I² = 28.5%, *P*=0.2368) (**Figure 3E**).

### 3.4 Safety outcomes of B7-H3-targeted ADCs

**Figure 4:**
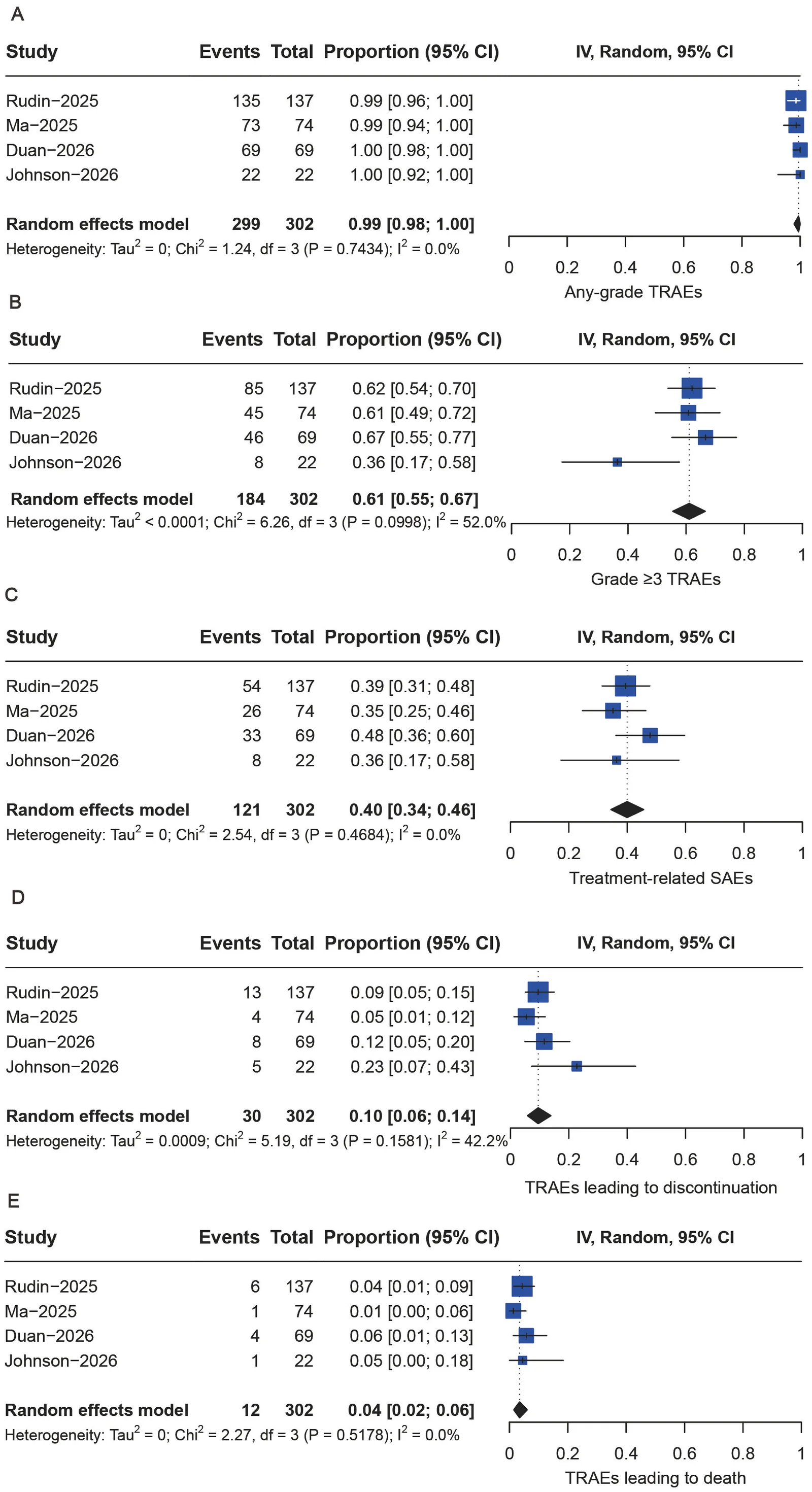
Forest plots of overall safety outcomes of B7-H3-targeted ADCs. **(A)** Any-grade TRAEs; **(B)** grade ≥3 TRAEs; **(C)** treatment-related SAEs; **(D)** TRAEs leading to treatment discontinuation; **(E)** TRAEs leading to death.

**Figure 5:**
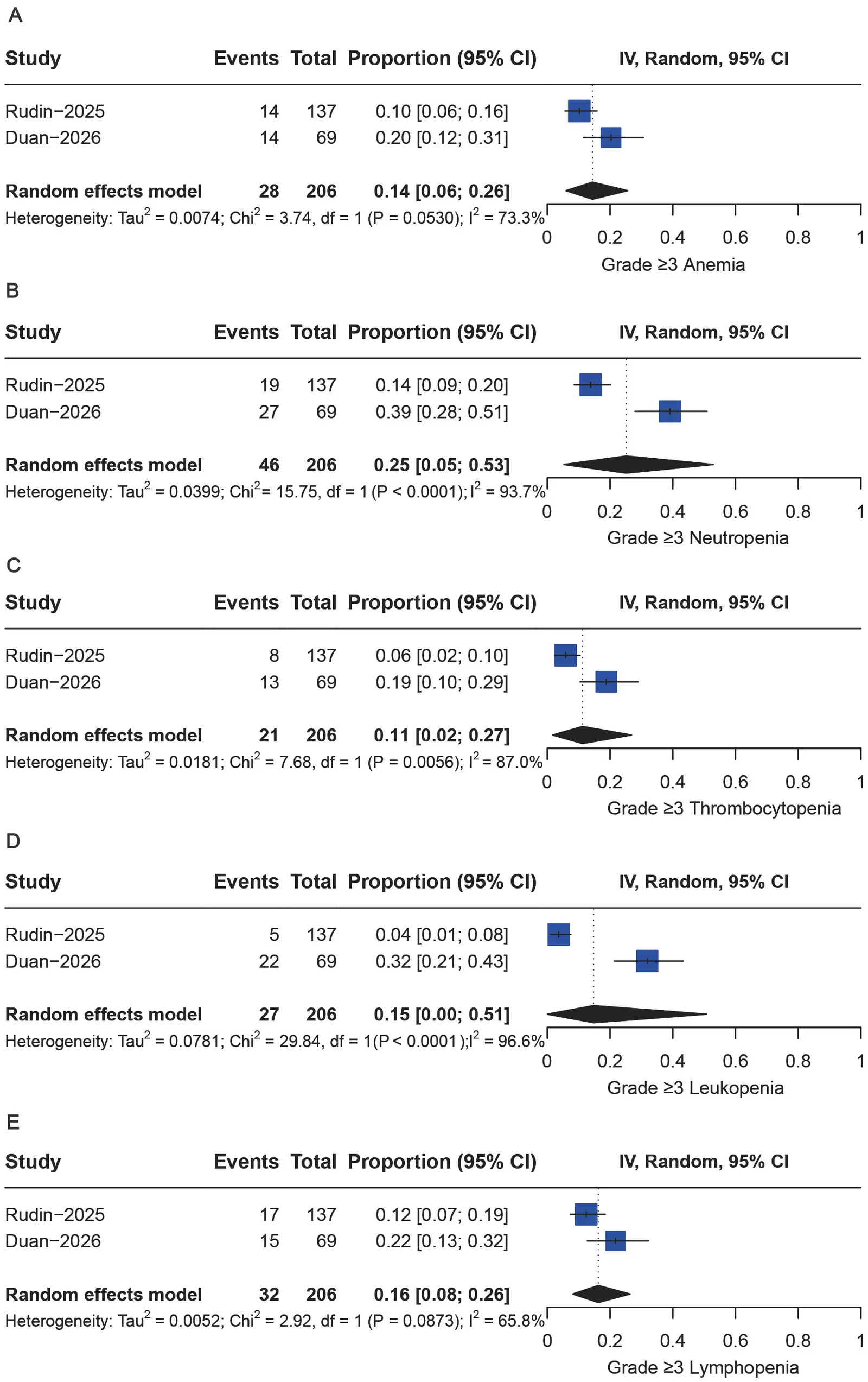
Forest plots of grade ≥3 hematologic toxicities associated with B7-H3-targeted ADCs. **(A)** Anemia; **(B)** neutropenia; **(C)** thrombocytopenia; **(D)** leukopenia; **(E)** lymphopenia.

**Figure 6:**
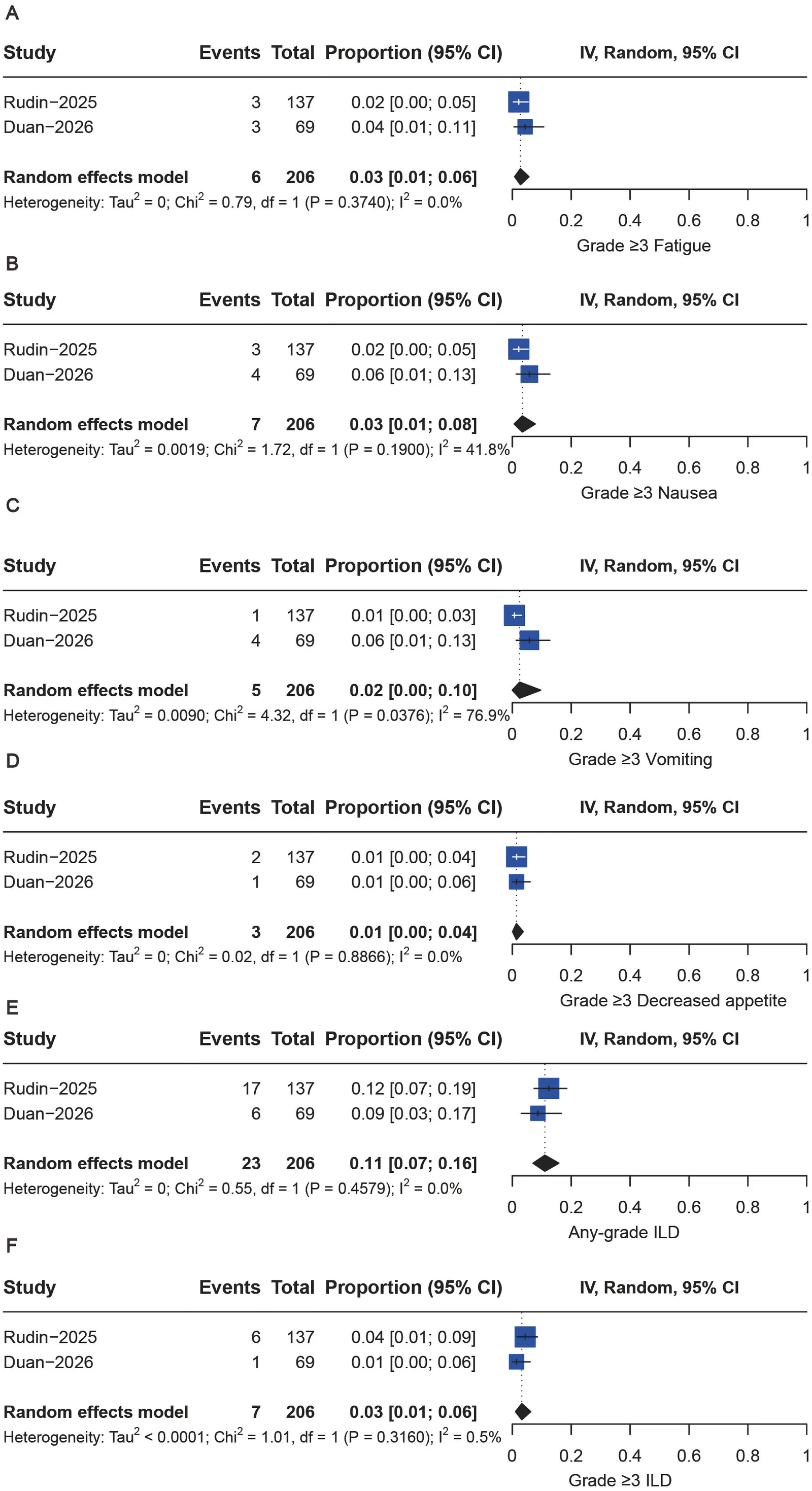
Forest plots of selected non-hematologic toxicities and ILD associated with B7-H3-targeted ADCs. **(A)** grade ≥3 fatigue; **(B)** grade ≥3 nausea; **(C)** grade ≥3 vomiting; **(D)** grade ≥3 decreased appetite; **(E)** any-grade ILD; **(F)** grade ≥3 ILD.

Four studies comprising 302 safety-evaluable patients were included in the overall safety analysis. The pooled incidence of any-grade treatment-related adverse events (TRAEs) was 99% (95% CI: 98%–100%, I² = 0.0%, *P*=0.7434), whereas grade ≥3 TRAEs occurred in 61% of patients (95% CI: 55%–67%, I² = 52.0%, *P*=0.0998) (**Figures 4A, B**). Treatment-related serious adverse events (SAEs) were reported in 40% of patients (95% CI: 34%–46%, I² = 0.0%, P=0.4684) (**Figure 4C**). TRAEs leading to treatment discontinuation or death occurred in 10% (95% CI: 6%–14%, I² = 42.2%, P=0.1581) and 4% (95% CI: 2%–6%, I² = 0.0%, P=0.5178) of patients, respectively (**Figures 4D, E**). Grade ≥3 hematologic toxicities were reported in two studies (Rudin-2025 and Duan-2026) comprising 206 patients. The pooled incidences of grade ≥3 anemia, neutropenia, thrombocytopenia, leukopenia, and lymphopenia were 14% (95% CI: 6%–26%, I² = 73.3%, *P*=0.0530), 25% (95% CI: 5%–53%, I² = 93.7%, *P*<0.0001), 11% (95% CI: 2%–27%, I² = 87.0%, *P*=0.0056), 15% (95% CI: 0%–51%, I² = 96.6%, *P*<0.0001), and 16% (95% CI: 8%–26%, I² = 65.8%, *P*=0.0873), respectively (**Figure 5**). Two studies (Rudin-2025 and Duan-2026) also reported grade ≥3 nonhematologic toxicities. The pooled incidences of fatigue, nausea, vomiting, and decreased appetite were 3% (95% CI: 1%–6%, I² = 0.0%, *P*=0.3740), 3% (95% CI: 1%–8%, I² = 41.8%, *P*=0.1900), 2% (95% CI: 0%–10%, I² = 76.9%, *P*=0.0376), and 1% (95% CI: 0%–4%, I² = 0.0%, *P*=0.8866), respectively (**Figures 6A–D**). Any-grade interstitial lung disease (ILD) occurred in 11% of patients (95% CI: 7%–16%, I² = 0.0%, *P*=0.4579), and grade ≥3 ILD occurred in 3% (95% CI: 1%–6%, I² = 0.5%, *P*=0.3160) (**Figures 6E, F**).

### 3.5 Exploratory contextual comparison of I-DXd and topotecan

**Figure 7:**
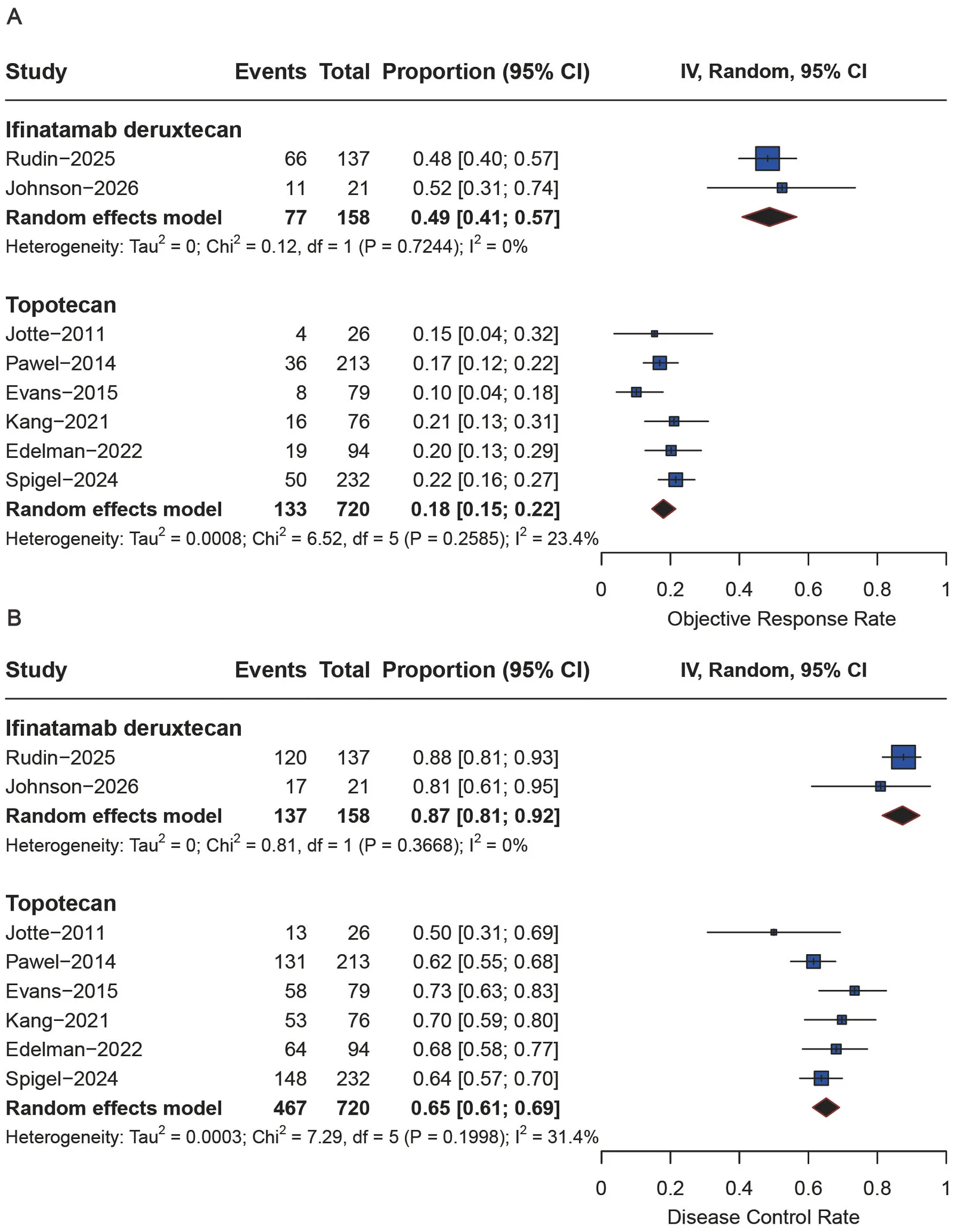
Forest plots providing a contextual comparison of tumor response outcomes between ifinatamab deruxtecan and topotecan. **(A)** ORR; **(B)** DCR.

**Figure 8:**
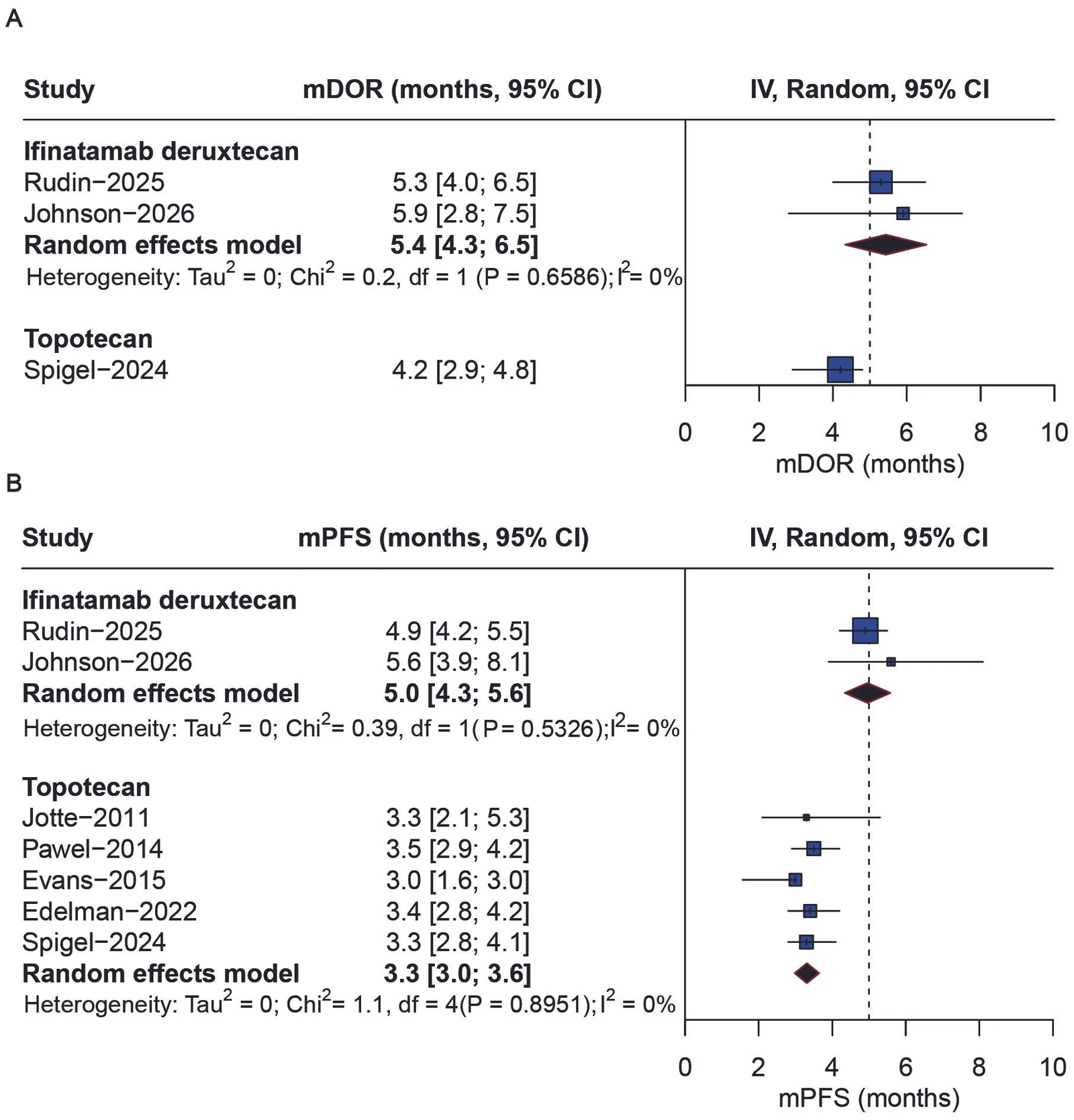
Forest plots providing a contextual comparison of time-to-event efficacy outcomes between ifinatamab deruxtecan and topotecan. **(A)** mDOR; **(B)** mPFS.

**Figure 9:**
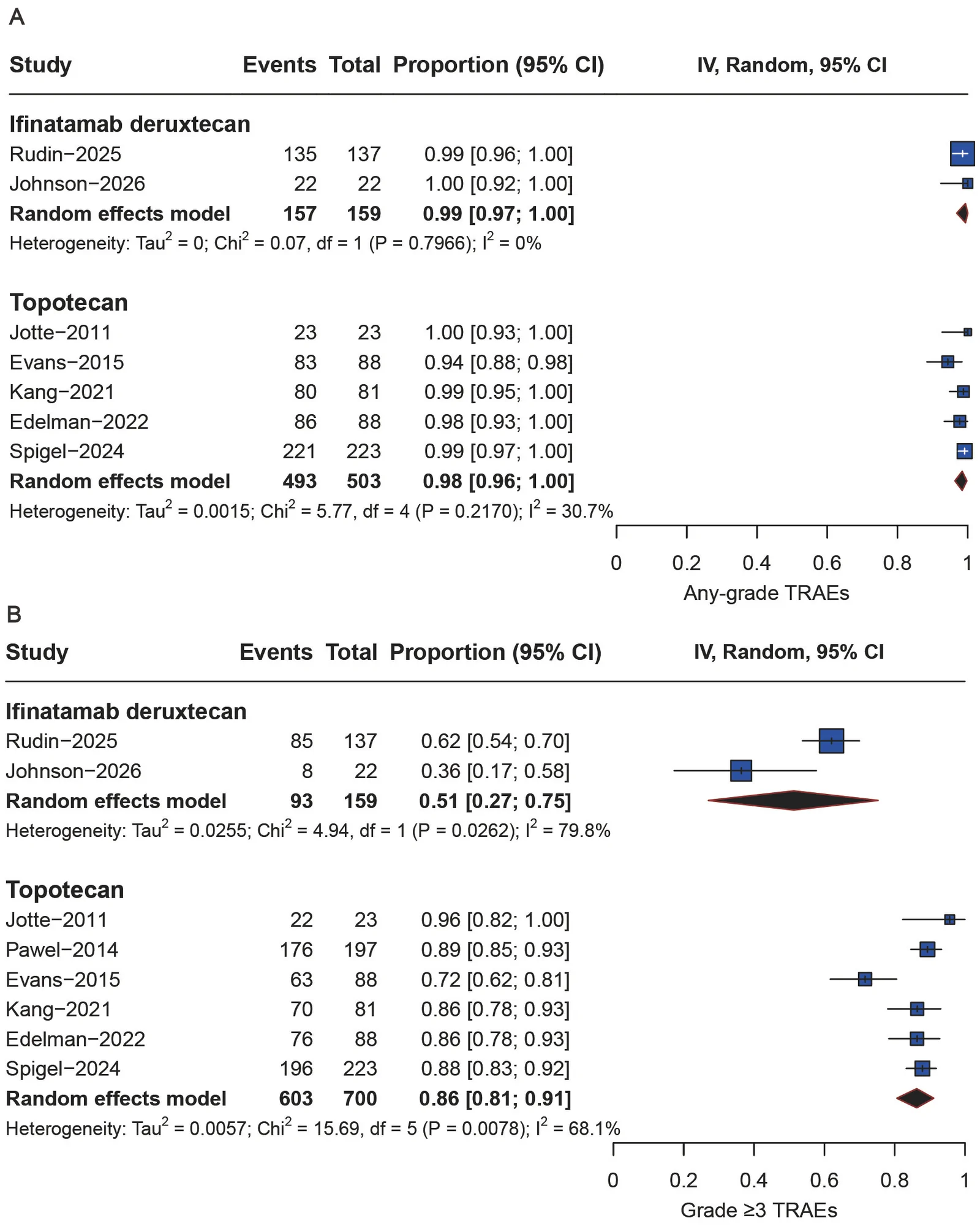
Exploratory contextual comparison of safety outcomes between ifinatamab deruxtecan and standard-dose intravenous topotecan. **(A)** Any-grade TRAEs; **(B)** grade ≥3 TRAEs.

For descriptive contextualization, outcomes for I-DXd and standard-dose IV topotecan were pooled separately and presented side by side. No formal between-group statistical comparisons were performed, and the reported P values refer to within-group heterogeneity. Two I-DXd (Rudin-2025 and Johnson-2026) studies comprising 158 efficacy-evaluable patients and six topotecan studies comprising 720 patients contributed to the ORR and DCR analyses. The pooled ORR was 49% for I-DXd (95% CI: 41%–57%, I² = 0.0%, P=0.7244) and 18% for topotecan (95% CI: 15%–22%, I² = 23.4%, P=0.2585), whereas the pooled DCR was 87% (95% CI: 81%–92%, I² = 0.0%, P=0.3668) and 65% (95% CI: 61%–69%, I² = 31.4%, P=0.1998), respectively (**Figure 7**). The pooled estimates for ORR and DCR were higher in the I-DXd studies than in the topotecan studies. The pooled mDOR and mPFS for I-DXd were 5.4 months (95% CI: 4.3–6.5 months, I² = 0.0%, P=0.6586) and 5.0 months (95% CI: 4.3–5.6 months, I² = 0.0%, P=0.5326), respectively. For topotecan, mDOR was available from one study (Spigel-2024) only (4.2 months, 95% CI: 2.9–4.8 months), whereas the pooled mPFS was 3.3 months (95% CI: 3.0–3.6 months, I² = 0.0%, P=0.8951) (**Figure 8**). The available time-to-event data showed longer mDOR and mPFS in the I-DXd studies; however, the mDOR estimate for topotecan was derived from a single study and should be interpreted with particular caution. Any-grade TRAEs occurred in 99% of patients receiving I-DXd (95% CI: 97%–100%, I² = 0.0%, P=0.7966) and 98% of those receiving topotecan (95% CI: 96%–100%, I² = 30.7%, P=0.2170), while grade ≥3 TRAEs occurred in 51% (95% CI: 27%–75%, I² = 79.8%, P=0.0262) and 86% (95% CI: 81%–91%, I² = 68.1%, P=0.0078), respectively (**Figure 9**). Any-grade TRAEs were similar between the two study groups, whereas the pooled incidence of grade ≥3 TRAEs was lower in the I-DXd studies. Substantial heterogeneity was observed for grade ≥3 TRAEs in both groups.

## 4 Discussion

This study provides a quantitative synthesis of the efficacy and safety of B7-H3-targeted ADCs in previously treated SCLC. The pooled ORR and DCR were 54% and 89%, respectively, while the pooled mDOR and mPFS were both 5.6 months. Given the aggressive nature of SCLC and the limited treatment options available after disease progression (42), these findings demonstrate encouraging and clinically meaningful antitumor activity and support the continued development of B7-H3-targeted ADCs in this setting. Nevertheless, the available evidence was derived from four early-phase single-arm studies, and the included ADCs differed in molecular design, dosing regimens, and enrolled patient populations. Future randomized studies are required to further define the clinical benefit of individual B7-H3-targeted ADCs and establish their optimal therapeutic positioning in relapsed SCLC. Grade ≥3 TRAEs and SAEs were also clinically relevant, underscoring the need for appropriate toxicity monitoring in future prospective studies.

Further analysis at the individual-agent level showed that representative B7-H3-targeted ADCs, including I-DXd, YL201, and HS-20093, demonstrated encouraging antitumor activity in their respective early-phase studies. The IDeate-Lung01 study (NCT05280470) evaluated I-DXd in patients with previously treated ES-SCLC, most of whom had previously received platinum-based chemotherapy with or without immunotherapy, and reported efficacy outcomes that were broadly consistent with the pooled estimates of the present meta-analysis (35). Although I-DXd, YL201, and HS-20093 all use IgG1 antibodies and topoisomerase I inhibitor payloads, their molecular structures differ substantially (**Table 4**). I-DXd combines the DXd payload with an Mc-Gly-Gly-Phe-Gly linker and has a drug-to-antibody ratio (DAR) of 4.0 (43), whereas YL201 incorporates a tumor-microenvironment-activatable tripeptide linker, the YL0010014 payload, and a higher DAR of 8.0. HS-20093 uses a Gly-Gly-Phe-Gly linker, the SHR-9265 payload, and a DAR of 4.0. These differences in linker design, payload, and DAR may influence systemic stability, intratumoral payload release, the bystander effect, antitumor activity, and toxicity (44, 45).

**Table 4:** Characteristics of B7-H3-targeted ADCs and topotecan included in the contextual analysis.

API	Company	Antibody	Linker	Payload	DAR
Ifinatamab deruxtecan	Daiichi Sankyo and Merck & Co., Inc.	IgG1	Mc-Gly-Gly-Phe-Gly	DXd(TOP1 inhibitor)	4.0
YL201	MediLink Therapeutics	IgG1	Tumor microenvironment-activatable tripeptide linker	YL0010014(TOP1 inhibitor)	8.0
HS-20093	Hansoh Pharma	IgG1	Gly-Gly-Phe-Gly	SHR-9265(TOP1 inhibitor)	4.0
Topotecan	GlaxoSmithKline (GSK)	NA	NA	NA	NA

Because I-DXd currently has the most mature clinical dataset among B7-H3-targeted ADCs, the exploratory contextual comparison with topotecan was restricted to I-DXd. In this exploratory contextual comparison, pooled estimates for ORR, DCR, mDOR, and mPFS were numerically higher for I-DXd than for topotecan. It should be noted that the interval to relapse and the closely related classification of platinum sensitivity are important prognostic factors in relapsed SCLC (46, 47). However, outcome data stratified by these variables were not consistently reported across the included studies and therefore could not be reliably harmonized or adjusted for at the study level. These differences may have contributed to the observed variation between treatment groups. Nevertheless, the consistent direction of all pooled efficacy outcomes in favor of I-DXd provides a clinically meaningful comparative signal and supports further evaluation of its clinical value relative to standard therapy in direct randomized controlled trials.

The observed antitumor activity is also biologically plausible given the role of B7-H3 in tumor progression. Functionally, B7-H3 exhibits dual roles in immunity: it was initially characterized as a co-stimulatory molecule promoting T-cell proliferation and IFN-γ production, whereas subsequent studies demonstrated co-inhibitory effects on T-cell activation and differentiation (48). Research has also demonstrated that B7-H3 contributes to regulating tumor cell proliferation, metastasis, metabolic reprogramming, and the emergence of drug resistance (49), and it is closely linked to unfavorable prognosis (50). These biological properties provide additional support for targeting B7-H3. Moreover, B7-H3 is expressed not only on tumor cells but also within the vasculature associated with tumors and specific stromal components (51). This indicates its potential role in influencing both tumor cells and the surrounding tumor microenvironment. For B7-H3-targeted ADCs incorporating cleavable linkers and membrane-permeable payloads, these expression characteristics may also provide a theoretical basis for the elimination of highly heterogeneous SCLC cell populations through a bystander effect (52). However, predictive biomarkers for B7-H3-targeted ADCs remain to be established. Most available early-phase studies did not require prospective patient selection according to B7-H3 expression, and the YL201 study found no significant association between baseline B7-H3 membrane expression and ORR (53). This finding does not exclude a potential predictive role for B7-H3 because differences in assay methodology, sampling site, spatial and temporal heterogeneity, and ADC-specific molecular design may affect the relationship between target expression and treatment response. Future studies should therefore incorporate standardized B7-H3 assays and prospective biomarker analyses to identify patients most likely to benefit.

Concerning safety, the factors contributing to toxicities related to ADCs are multifactorial and may include premature payload release into the systemic circulation (54), chemical instability of the linker (55), inefficient tumor delivery caused by barriers within the tumor microenvironment, and on-target/off-tumor effects resulting from antigen expression in normal tissues (56). These mechanisms may collectively influence both systemic toxicity and the therapeutic window of ADCs. In the present analysis, the pooled incidences of any-grade TRAEs, grade ≥3 TRAEs, and SAEs were 99%, 61%, and 40%, respectively. The pooled incidences of TRAEs leading to treatment discontinuation and death were 10% and 4%, respectively. These findings highlight the importance of careful toxicity management during further clinical development. Common toxicities mainly included hematologic adverse events, gastrointestinal symptoms and fatigue, which were generally managed in the included studies through dose adjustment, symptomatic treatment, and supportive care (57, 58). Although ILD was less frequent than hematologic and gastrointestinal toxicities, it remains an important agent-specific safety concern. Notably, in the phase II IDeate-Lung01 trial, adjudicated treatment-related ILD occurred in 12.4% of patients receiving I-DXd at 12 mg/kg, including grade ≥3 events in 4.4% (59), highlighting the need for agent-specific pulmonary monitoring. Safety monitoring should therefore be tailored to the toxicity profile of each ADC. Before broader clinical implementation, further optimization of dosing schedules and intervals is needed, together with agent-specific monitoring for myelosuppression, gastrointestinal toxicity, and ILD and predefined criteria for dose modification and treatment discontinuation.

When interpreted in the context of the current treatment landscape for SCLC, our findings suggest that B7-H3-targeted ADCs may provide a potential option in later-line therapy, although their clinical positioning must account for the emergence of tarlatamab as a new second-line standard. In the phase III DeLLphi-304 trial (NCT05740566), the DLL3-targeted bispecific T-cell engager tarlatamab significantly prolonged median overall survival compared with investigator’s-choice chemotherapy (13.6 versus 8.3 months; HR, 0.60) and also improved median PFS (4.2 versus 3.7 months; HR, 0.71) (8). Unlike tarlatamab, whose clinical benefit is supported by randomized phase III evidence, current evidence for B7-H3-targeted ADCs is derived predominantly from early-phase, single-arm studies. Furthermore, the pooled mDOR and mPFS of 5.6 months in the present analysis indicate that the high ORR should be interpreted together with response durability and disease control rather than in isolation. Tarlatamab is associated particularly with cytokine release syndrome and neurologic toxicities, whereas B7-H3-targeted ADCs are characterized mainly by hematologic and gastrointestinal toxicities, together with agent-specific risks such as ILD. However, in the absence of direct comparative evidence, these differences cannot establish that either therapeutic class has a more favorable overall safety profile. Accordingly, current evidence supports tarlatamab as the second-line standard for eligible patients, whereas B7-H3-targeted ADCs may be investigated after progression on or intolerance to tarlatamab or in patients for whom the management of immune effector cell-related toxicities is unsuitable, preferably within clinical trials. This proposed sequence remains hypothesis-generating because no prospective sequencing studies have been reported. The distinct mechanisms of action and partially non-overlapping toxicity profiles of these treatments also provide a rationale for prospective evaluation of sequential and combination strategies, although their efficacy and safety require formal investigation (48).

Although clinical evidence regarding B7-H3-targeted ADCs in SCLC remains immature, the data available thus far indicate promising antitumor effects and a generally manageable safety profile, which supports the continued development of these agents as candidates with the potential to achieve a favorable balance between efficacy and safety. Several phase III trials are currently evaluating B7-H3-targeted ADCs in SCLC, including IDeate-Lung02 for I-DXd (NCT06203210) (60), ARTEMIS-008 for HS-20093 (61), and a phase III trial of YL201 (NCT06612151) (62). The results of these ongoing trials will be important for defining their optimal dosing strategies, identifying the patient populations most likely to benefit, and clarifying their relative clinical value compared with existing standard later-line therapies.

## 5 Conclusion

In conclusion, this study represents the first meta-analysis to systematically synthesize the efficacy and safety profile of B7-H3-targeted ADCs in SCLC. The pooled findings suggest that these agents demonstrate relatively high ORR and DCR in patients with previously treated SCLC. In the exploratory contextual comparison, I-DXd demonstrated numerically favorable efficacy estimates and selected safety outcomes compared with standard-dose IV topotecan. The current analysis provides additional evidence supporting the antitumor activity of B7-H3-targeted ADCs in SCLC, highlights the potential clinical relevance of B7-H3 as a therapeutic target, and provides a rationale for future phase III studies and prospective evaluation of sequential and combination strategies.

## Data Availability

The original contributions presented in the study are included in the article/**Supplementary Material**. Further inquiries can be directed to the corresponding authors.
