# Supplementary material for: Efficacy and safety of B7-H3-targeted antibody-drug conjugates in previously treated small cell lung cancer: a systematic review and meta-analysis with a contextual comparison to topotecan
Source: Front Immunol. 2026 Aug 13;17:1907324. doi: 10.3389/fimmu.2026.1907324 (PMC13518547; doi:10.3389/fimmu.2026.1907324)
Supplement: Supplementary file 1 [file Table2.docx]

Supplementary Table 2. Characteristics of potentially relevant B7-H3-targeted ADC studies and reasons for exclusion.

| No. | Drug | NCT | Phase | Available report | Decision | Reason |
| --- | --- | --- | --- | --- | --- | --- |
| 1 | MHB088C | CTR20231298 | I/II | Conference abstract | Excluded | Only a conference abstract was available; no full-text original article had been published by the search cutoff. |
| 2 | 7MW3711 | [NCT06008366](https://clinicaltrials.gov/study/NCT06008366) | I/II | Conference abstract | Excluded | Only a conference abstract was available; no full-text original article had been published by the search cutoff. |
| 3 | DB1311/BNT324 | NCT05914116 | I/II | Conference abstract | Excluded | Only a conference abstract was available; no full-text original article had been published by the search cutoff. |
| 4 | SYS6043 | NCT07415863 | I/II | Conference abstract | Excluded | Only a conference abstract was available; no full-text original article had been published by the search cutoff. |
| 5 | HS-20093 | NCT07099898 | III | Conference abstract | Excluded | Only a conference abstract was available; no full-text original article had been published by the search cutoff. |
