# Supplementary material for: Efficacy and safety of B7-H3-targeted antibody-drug conjugates in previously treated small cell lung cancer: a systematic review and meta-analysis with a contextual comparison to topotecan
Source: Front Immunol. 2026 Aug 13;17:1907324. doi: 10.3389/fimmu.2026.1907324 (PMC13518547; doi:10.3389/fimmu.2026.1907324)
Supplement: Supplementary file 2 [file Table1.doc]

**Supplementary Table 1. The detailed search strategy.**

| Databases | Set | Search terms |
| --- | --- | --- |
| **Pubmed** | #1 | "Small Cell Lung Carcinoma"[MeSH Terms] OR "carcinoma small cell lung"[Title/Abstract] OR "oat cell carcinoma of lung"[Title/Abstract] OR "oat cell lung cancer"[Title/Abstract] OR "small cell cancer of the lung"[Title/Abstract] OR "small cell lung cancer"[Title/Abstract] |
|  | #2 | ("Topotecan"[Title/Abstract] OR "9 dimethylaminomethyl 10 hydroxycamptothecin"[Title/Abstract] OR "9 dimethylaminomethyl 10 hydroxycamptothecin"[Title/Abstract] OR "Topotecan Hydrochloride"[Title/Abstract] OR "hydrochloride topotecan"[Title/Abstract] OR "Nogitecan Hydrochloride"[Title/Abstract] OR "Hycamtin"[Title/Abstract] OR "nsc 609699"[Title/Abstract] OR "NSC609699"[Title/Abstract] OR "nsc 609699"[Title/Abstract] OR "skf 104864 a"[Title/Abstract] OR "skf 104864 a"[Title/Abstract]) AND (randomizedcontrolledtrial[Filter]) |
|  | #3 | #1 AND #2 |
|  | #4 | "antibody drug conjugate"[Title/Abstract] OR "antibody drug conjugate"[Title/Abstract] OR "ADC"[Title/Abstract] |
|  | #5 | "CD276"[Title/Abstract] OR "b7 homolog 3"[Title/Abstract] OR "B7-H3"[Title/Abstract] OR "B7H3"[Title/Abstract] OR "immunoregulatory protein b7 homologue 3"[Title/Abstract] OR "4Ig-B7-H3"[Title/Abstract] OR "B7RP-2"[Title/Abstract] |
|  | #6 | #4 AND #5 |
|  | #7 | #1 AND #6 |
| **Embase** | #1 | 'small cell lung carcinoma':ab,ti OR 'carcinoma, small cell lung':ab,ti OR 'oat cell carcinoma of lung':ab,ti OR 'oat cell lung cancer':ab,ti OR 'small cell cancer of the lung':ab,ti OR 'small cell lung cancer':ab,ti |
|  | #2 | 'topotecan'/exp OR '10 [ (dimethylamino) methyl] 4 ethyl 4, 9 dihydroxy 1h pyrano [3`, 4`:6, 7] indolizino [1, 2 b] quinoline 3, 14 (4h, 12h) dione':ti,ab,kw OR '8 [ (dimethylamino) methyl] 19 ethyl 7, 19 dihydroxy 17 oxa 3, 13 diazapentacyclo [11.8.0.0 (2, 11) .0 (4, 9) .0 (15, 20)] henicosa 1 (21), 2, 4 (9), 5, 7, 10, 15 (20) heptaene 14, 18 dione':ti,ab,kw OR '9 dimethylaminomethyl 10 hydroxycamptothecin':ti,ab,kw OR 'alpha, 8 dihydroxy 1 (11h) oxo alpha ethyl 2 (hydroxymethyl) 9 (dimethylaminomethyl) 5, 11a diaza 11ah benzo [b] fluorene 3 acetic acid 3, 2 lactone':ti,ab,kw OR 'e 89001':ti,ab,kw OR e89001:ti,ab,kw OR evotopin:ti,ab,kw OR 'ff 10850':ti,ab,kw OR ff10850:ti,ab,kw OR hycamptamine:ti,ab,kw OR hycamtin:ti,ab,kw OR lutecan:ti,ab,kw OR 'nk 211':ti,ab,kw OR nk211:ti,ab,kw OR nogitecan:ti,ab,kw OR 'nsc 609699':ti,ab,kw OR nsc609699:ti,ab,kw OR oncotecan:ti,ab,kw OR potactasol:ti,ab,kw OR ribocamtin:ti,ab,kw OR 'skf 104864':ti,ab,kw OR 'skf 104864 a':ti,ab,kw OR 'skf 104864a':ti,ab,kw OR 'skf s 104864a':ti,ab,kw OR 'skf s104864a':ti,ab,kw OR skf104864:ti,ab,kw OR skf104864a:ti,ab,kw OR topocan:ti,ab,kw OR topoliquid:ti,ab,kw OR 'topotecan hydrochloride':ti,ab,kw OR topotecane:ti,ab,kw OR topotecano:ti,ab,kw OR topotekan:ti,ab,kw OR topotel:ti,ab,kw OR topovin:ti,ab,kw OR topotecan:ti,ab,kw |
|  | #3 | #1 AND #2 |
|  | #4 | 'antibody-drug conjugate':ab,ti OR 'antibody drug conjugate':ab,ti OR adc:ab,ti |
|  | #5 | cd276:ab,ti OR 'b7 homolog 3':ab,ti OR 'b7 h3':ab,ti OR 'b7h3 protein':ab,ti |
|  | #6 | #4 AND #5 |
|  | #7 | #1 AND #6 |
| **Cochrane**  **Library** | #1 | ("small cell lung carcinoma"):ti,ab,kw OR ("Carcinoma, Small Cell Lung"):ti,ab,kw OR ("Oat Cell Carcinoma of Lung"):ti,ab,kw OR ("Oat Cell Lung Cancer"):ti,ab,kw OR ("Small Cell Cancer Of The Lung"):ti,ab,kw |
|  | #2 | MeSH descriptor: [Topotecan] explode all trees |
|  | #3 | (topotecan* OR nogitecan* OR hycamptamin* OR Hycamtin OR Hycamptin OR "9 dimethylaminomethyl 10 hydroxycamptothecin" OR SKF104864A OR "SKF 104864A" OR "SKF 104864 A" OR "SKF-104864-A" OR SKF104864 OR "SKF 104864" OR NSC609699 OR "NSC-609699"):ti,ab,kw |
|  | #4 | #2 OR #3 |
|  | #5 | #1 AND #4 |
|  | #6 | (CD276):ti,ab,kw OR (B7 homolog 3):ti,ab,kw OR (B7H3):ti,ab,kw |
|  | #7 | (antibody-drug conjugate):ti,ab,kw OR (Antibody drug conjugate):ti,ab,kw OR (ADC):ti,ab,kw |
|  | #8 | #6 AND #7 |
|  | #9 | #1 AND #8 |
